# Association Between Arterial Stiffness and Fatigability in Well-Functioning Older Adults

**DOI:** 10.1093/geroni/igab046.798

**Published:** 2021-12-17

**Authors:** Ajoy Karikkineth, Luigi Ferrucci, Eleanor Simonsick

**Affiliations:** 1 National Institute on Aging, Baltimore, Maryland, United States; 2 National Instute on Aging/NIH, Baltimore, Maryland, United States

## Abstract

The association between vascular health measured by arterial stiffness and fatigability, a marker of future mobility decline, is unknown. We examined 1210 men (47.7%) and women from the Baltimore Longitudinal Study of Aging, mean age 66.6 ± 13.9 years. Perceived fatigability was assessed after a 5-minute, treadmill walk using Borg rating (range 6-20). Arterial stiffness was determined by carotid femoral pulse wave velocity (PWV). In linear regression analyses fatigability and PWV were associated in men (Beta/P-value) (0.160/0.001) and women (0.136/0.008). Adjustment for mean arterial and pulse pressure attenuated the association in women (0.104/0.050) but not men (0.160/0.001). The association was significant among those with slower usual and rapid gait speeds, longer 400m walk time and slower repeated chair stands pace (all p<0.05). Arterial stiffness is associated with a greater proneness to fatigue especially in older adults exhibiting poorer mobility. The underlying mechanisms appear to differ between men and women.

